# The effect of three-layer liner on the jet formation and penetration capability of shaped charge jet

**DOI:** 10.1038/s41598-023-38680-y

**Published:** 2023-08-24

**Authors:** Zhiwei Hao, Zhijun Wang, Yifan Wang, Conghui Duan, Qing Ji

**Affiliations:** College of Mechanical and Electrical Engineering, North University of China, Taiyuan, 030051 China

**Keywords:** Engineering, Materials science

## Abstract

In order to solve the problem of insufficient penetration ability of common liner, a new three-layer liner was proposed. AUTODYN software was used to simulate the efflux forming process of three-layer liner. The influence of four different impact impedance liner materials and different thickness ratio of three-layer liner on efflux performance was studied. In order to study the penetration ability of shaped charge to semi-infinite target plate, the penetration calculation of the target plate was carried out by taking several standoff under different thickness ratios. The optimal thickness ratio of three-layer liner was determined by studying the penetration depth and opening size of the target plate. The results show: By comparing the matching of liner materials with different impact impedances, the head velocity, effective length, total energy, total kinetic energy and effective jet mass of the jet formed when the three-layer liner material is AL 2024, copper and nickel from the outside to the inside are the best. In the analysis of the matching of the thickness ratio of the three-layer liner, the key to the jet forming is the thickness ratio of the outer liner. Within a certain range, the greater the proportion of the thickness of the outer liner, the better the jet forming; when the material of the three-layer liner is AL 2024-copper-nickel from outside to inside, the thickness ratio of the liner is 4/1/1 from outside to inside, and the jet forming is the best. The maximum penetration depth of the shaped charge with a thickness ratio of 1/1/4 from the outside to the inside of the three-layer liner is 395.5 mm, which is 52.3% higher than that of the shaped charge with a double-layer liner. Compared with the shaped charge with single-layer liner, the penetration depth is increased by 62.6%. When the thickness ratio of the three-layer liner is 1/1/4 from the outside to the inside, the maximum entrance diameter of the target plate is 14.3 mm, which is the same as that of the shaped charge with the double-layer liner. Compared with the shaped charge with single-layer liner, the entrance diameter is in-creased by 14.4%.

## Introduction

At present, shaped charge technology has been widely used in military and civilian fields. Firstly, in the military field, shaped charge technology is widely used in various types of anti-tank and anti-armor warheads. Secondly, in the civil field, mining, tunnel excavation and so on can use the energy gathering effect of explosives^1–3^.

The shaped charge has a series of advantages such as relatively simple launch platform, high energy utilization rate and strong penetration ability, and has played a huge role in military and civilian fields. In recent years, with the development of modern science and technology, the protection ability of various new types of armor has been continuously improved. As one of the effective anti-armor means, the shaped charge armor-piercing ammunition is limited by the traditional shaped charge formation mechanism and the density, sound speed and ductility of the liner material itself. Its armor-piercing ability is difficult to achieve efficient damage to high-strength armor protection targets. Therefore, it is very important and urgent to study the new shaped charge structure^4^.

Therefore, a new type of three-layer liner structure is designed in this paper. Compared with the traditional single-layer liner shaped charge and double-layer liner shaped charge, the three-layer liner shaped charge has many advantages, such as large jet head speed and large jet stretch length. However, from the current data, there is no systematic study on the jet formed by the three-layer liner. The core problem is to study the mechanism of the collapse of the inner and outer liner after the action of detonation products and the mechanism of converging into the jet at the pressure span point. This involves the matching of the impact impedance of the inner and outer liner, the influence of the thickness and the initiation process on the stress wave transmission. In this paper, four kinds of liner materials with different impact impedance, Al 2024, copper, nickel and tantalum, are used to study the best jet under different material matching. In addition, the damage of semi-infinite target plate by different standoff under different thickness ratios of three-layer liner is studied, and the optimal thickness ratio of three-layer liner is obtained. This is of great academic value for improving the penetration ability of jet and improving the utilization rate of jet^5–9^.

## Numerical calculation model

### Establish numerical calculation model

**Figure 1:**
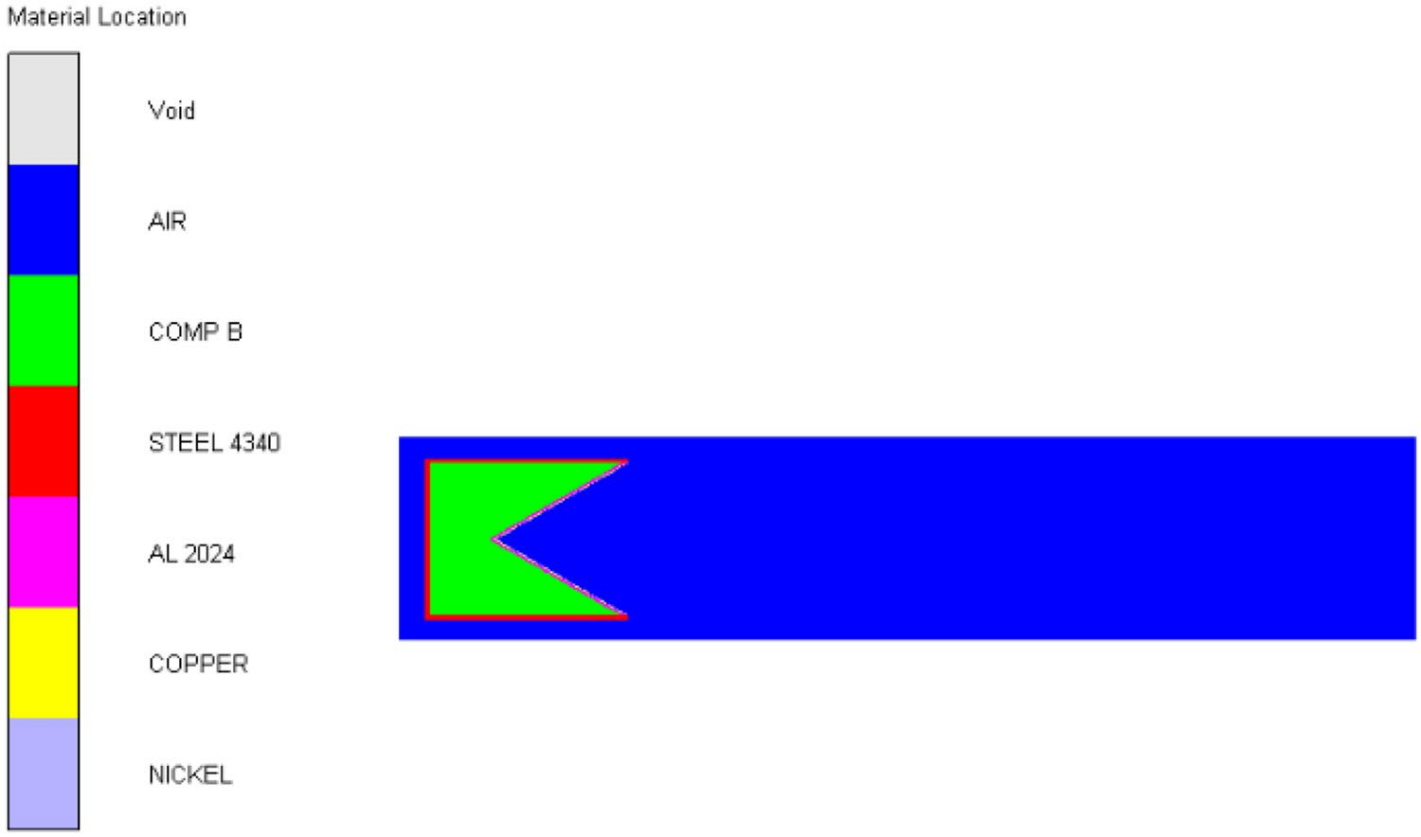
Finite difference model of shaped charge.

**Figure 2:**
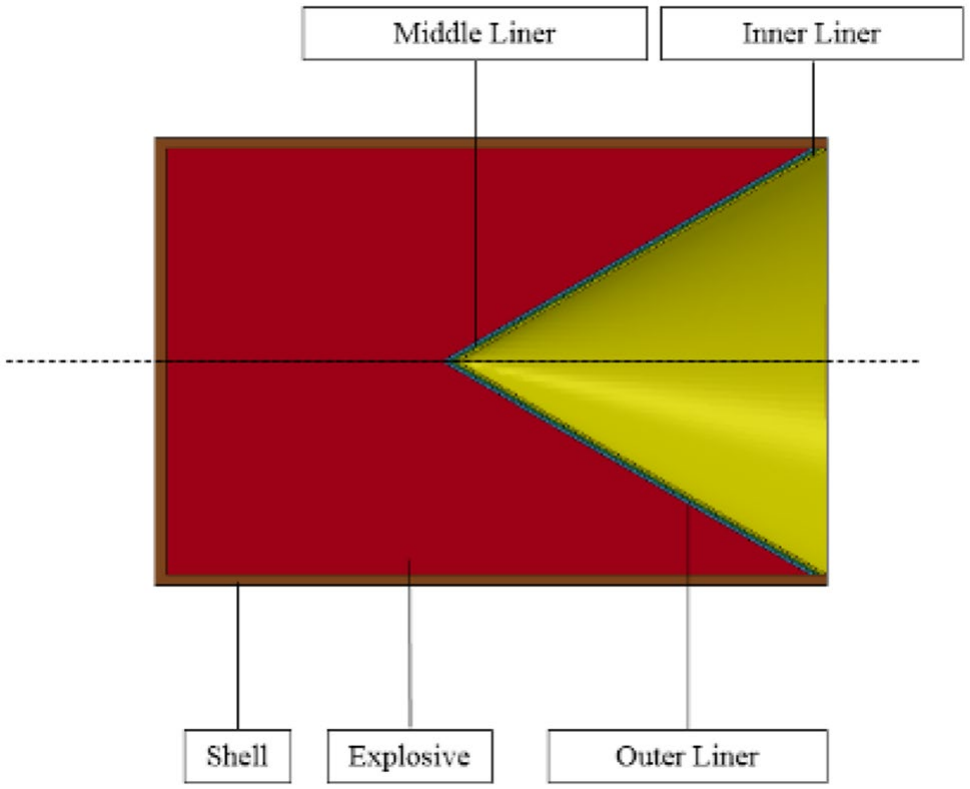
Structure diagram of shaped charge.

The finite difference model of the three-layer liner structure is shown in Fig. 1. The structural parameters of the warhead are as follows: the main charge length is 78 mm, the charge diameter is 60 mm, the cone angle of the liner is 60°, the shell thickness is 2 mm, and the total thickness of the three-layer liner is 3 mm. The detonation mode is initiated by the bottom center of the explosive, and the warhead structure is shown in Fig. 2.

Because the shaped charge structure is axisymmetric, in order to reduce to calculation time, a two-dimensional axisymmetric model is established. The model adopts the mm-mg-ms unit system. The numerical model in this paper is composed of air, shell, explosive, inner liner, middle liner, outer liner and target plate. Because the jet forming process is a large deformation problem, the air, shell, explosive and liner adopt Euler algorithm. The ' FLOW OUT ' boundary condition is added to the boundary of the air domain to prevent the reflection of the detonation products, and the Euler mesh size is 0.25 mm × 0.25 mm.

### Material model

The materials are selected from the material library of AUTODYN-2D simulation software. The material models and parameters of shaped charge warhead and target plate are shown in Table 1:

**Table 1:** Material model and parameters.

Component	Materials	EOS	Strength	Failure
AIR	AIR	Ideal Gas	None	None
Explosive	COMP B	JWL	None	None
Shell	STEEL 4340	Linear	Johnson Cook	None
Target	STEEL 45	Linear	Johnson Cook	None
Liner	AL 2024	Shock	None	None
Liner	COPPER	Shock	None	None
Liner	NICKEL	Shock	None	None
Liner	TANTALUM	Shock	None	None

The equation of state for air (EOS) is an ideal gas, and it can be used in many applications involving the motion of gases. This equation can be derived from Boyle's law and Gay-Lussac's law, and it is expressed as^5–7^:

1 $${\mathrm{P}}_{\mathrm{A}}=(\upgamma -1){\uprho }_{\mathrm{A}}{\mathrm{E}}_{\mathrm{A}}$$

In the equation, ${\uprho }_{\mathrm{A}}$ is the air density of $1.225\times {10}^{-3}\mathrm{ g}/{\mathrm{cm}}^{3}$; $\upgamma$ as gas constant, is 1.4; ${\mathrm{E}}_{\mathrm{A}}$ is 206.8 $\mathrm{kJ}/{\mathrm{m}}^{3}$.

Detonation model of high energy explosive is selected for shaped charge material, and the equation of state is JWL equation of state, whose basic form is:

2 $$\mathrm{P}=\mathrm{A}\left(1-\frac{\upomega }{{\mathrm{R}}_{1}\mathrm{V}}\right){\mathrm{e}}^{-{\mathrm{R}}_{1}\mathrm{V}}+\mathrm{B}\left(1-\frac{\upomega }{{\mathrm{R}}_{2}\mathrm{V}}\right){\mathrm{e}}^{-{\mathrm{R}}_{2}\mathrm{V}}+\frac{\mathrm{\omega E}}{\mathrm{V}}$$

In formula (1): A, B, ${\mathrm{R}}_{1}$, ${\mathrm{R}}_{2}$, $\upomega$ are input parameters; P, E and V are respectively the pressure of explosive products, the internal energy per unit volume and the relative volume (the volume of detonation products produced by the charge per unit volume). Specific parameters are shown in Table 2 below.

**Table 2:** Material parameters of B explosive.

$\rho \;({\text{g}}/{\text{cm}}^{3} )$	${\text{E}}\;{\text{(kJ}} \cdot {\text{m}}^{ - 3} )$	${\text{A}}\;({\text{Gpa)}}$	${\text{B}}\;({\text{Gpa)}}$	${\text{R}}_{1}$	${\text{R}}_{2}$	$\omega$
1.717	$8.50\times {10}^{6}$	524.23	7.678	4.2	1.1	0.34

For the STEEL 4340 used in the shell, EOS is a linear model and the strength model is described by the Johnson–Cook equation, where the yield stress Y is defined as:

3 $$\mathrm{Y}=(\mathrm{A}+\mathrm{B}{\upvarepsilon }_{\mathrm{P}}^{\mathrm{n}})(1+\mathrm{Clog}{\upvarepsilon }_{\mathrm{P}}^{*})(1-{\mathrm{T}}_{\mathrm{H}}^{\mathrm{m}})$$

where ${\upvarepsilon }_{\mathrm{P}}$ is the effective plastic strain, A, B, C, n and m for constant,${\mathrm{E}}_{\mathrm{P}}^{*}={\upvarepsilon }_{\mathrm{P}}^{\cdot}/{\upvarepsilon }_{0}^{\cdot}$ with ${\upvarepsilon }_{0}^{\cdot}=1 {\mathrm{s}}^{-1}$ is the standardized effective plastic strain rate. ${\mathrm{T}}_{\mathrm{H}}^{\mathrm{m}}$ is the homologous temperature, and the calculation formula is:

4 $${\mathrm{T}}_{\mathrm{H}}^{\mathrm{m}}=(\mathrm{T}-{\mathrm{T}}_{\mathrm{room}})/({\mathrm{T}}_{\mathrm{melt}}-{\mathrm{T}}_{\mathrm{room}})$$

where ${\mathrm{T}}_{\mathrm{melt}}$ is the melting temperature and ${\mathrm{T}}_{\mathrm{room}}$ is the room temperature. The specific parameters are shown in Table 3 below:

**Table 3:** Parameters in the strength model of STEEL 4340.

A (Mpa)	B (Mpa)	n	C	m	${\mathrm{T}}_{\mathrm{melt}}$ (K)	${\mathrm{T}}_{\mathrm{room}}$ (K)
760.0	507.0	0.28	0.064	1.06	1793.0	300.0

For the STEEL 45 used in the target plate, EOS is a linear model, and the strength model is described by Johnson–Cook equation. The specific parameters are shown in Table 4:

**Table 4:** Material parameters of STEEL 45.

Materials	G/GPa	${\mathrm{f}}_{\mathrm{c}}$/GPa	${\mathrm{f}}_{\mathrm{t}}$/${\mathrm{f}}_{\mathrm{c}}$	${\mathrm{f}}_{\mathrm{s}}$/${\mathrm{f}}_{\mathrm{c}}$	A	N	${\mathrm{Q}}_{2}$	B	M	${\mathrm{D}}_{1}$	${\mathrm{D}}_{2}$
Concrete	16.7	35	0.1	0.18	1.6	0.61	0.68	1.6	0.61	0.04	1.0

## Analysis of impact impedance of different materials on jet forming

In order to study the influence of the impact impedance of the material on the jet forming, the numerical simulation is carried out when the structural parameters of the shaped charge are consistent and the thickness of the outer liner, the middle liner and the inner liner of the three-layer liner is 1 mm.

The propagation velocity of detonation wave in the medium is closely related to the impact impedance of the material. The inner metal of the liner is the main part of the jet, and the pestle body is mainly composed of outer metal. The inner liner adopts materials with high density, good plasticity and easy formation of jet, while the outer liner adopts materials with low density and low impedance. On the premise of ensuring that the inner metal mass is sufficient to form a penetrating jet, the overall density of the liner can be reduced, and the outer material can play the role of transmitting and increasing pressure. In order to make the jet have better penetration performance, the choice of liner material should be based on this rule^9–11^. In this section, AL 2024, COPPER, NICKEL and TANTALUM were selected as the material of the three-layer liner. It can be seen from the literature^9^ that the impact impedance of the material can be approximately expressed by $\mathrm{R}=\mathrm{\rho c}$. Where, c is the sound speed of the material. See Table 5 for the specific parameters of the material. The material scheme for numerical simulation of jet flow from three-layer liner is shown in Table 6.

**Table 5:** Basic parameters of different materials.

Materials	Density ($\mathrm{g}/{\mathrm{cm}}^{3}$)	sound velocity (m/s)	Impedance of impact ($\mathrm{N}\cdot s/{\mathrm{m}}^{3}$)
AL 2024	2.785	5328	$1.48\times {10}^{7}$
COPPER	8.93	3940	$3.52\times {10}^{7}$
NICKEL	8.874	4602	$4.08\times {10}^{7}$
TANTALUM	16.654	3414	$5.69\times {10}^{7}$

**Table 6:** Material matching scheme of three-layer liner.

Scheme	Outer liner	Middle liner	Inner liner
1	AL 2024	COPPER	NICKEL
2	AL 2024	COPPER	TANTALUM
3	AL 2024	NICKEL	TANTALUM
4	COPPER	NICKEL	TANTALUM

**Figure 3:**
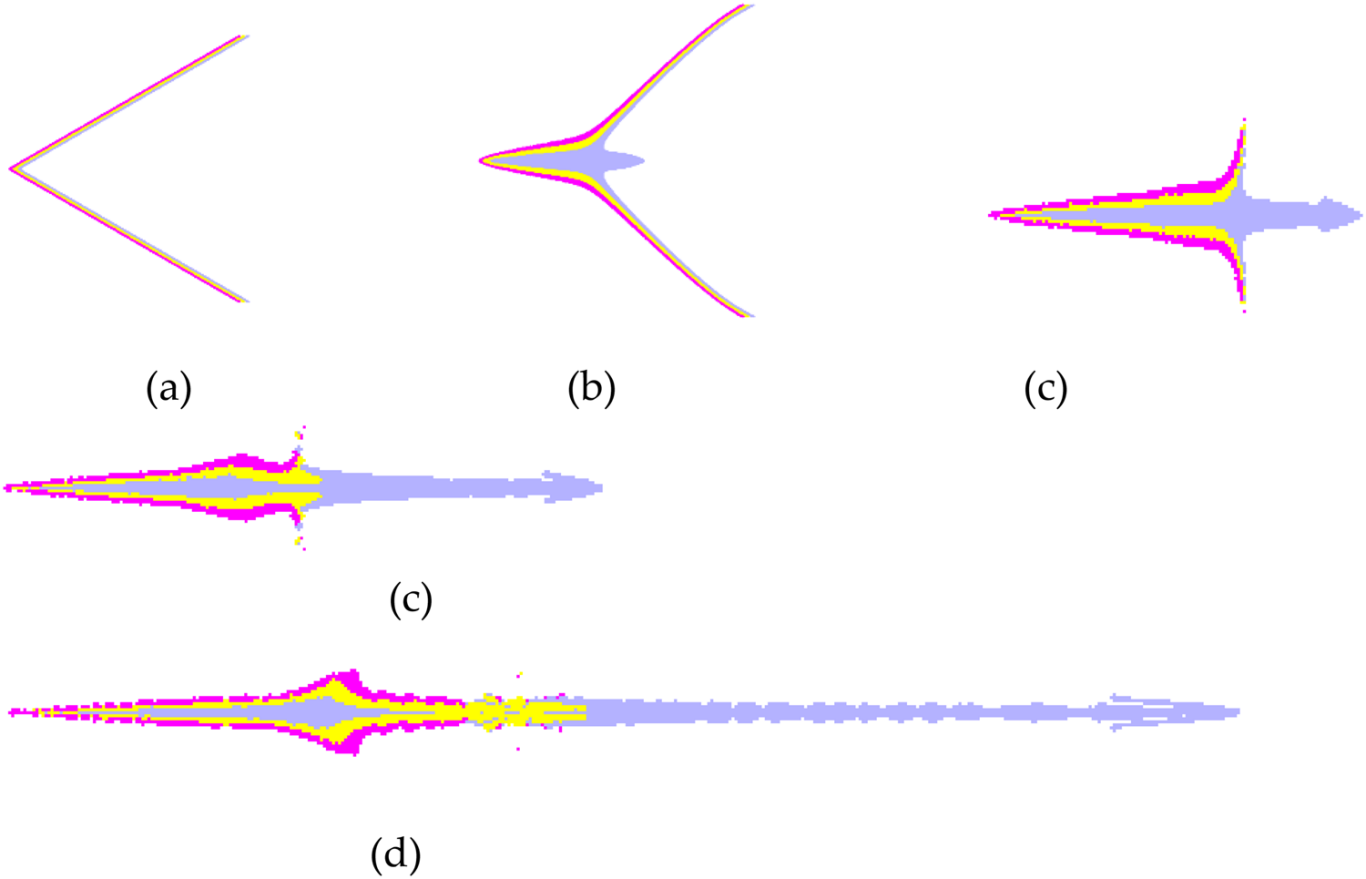
Schematic diagram of jet forming process of three-layer liner. (**a**) t = 0 $\mathrm{\mu s}$. (**b**) t = 10 $\mathrm{\mu s}.$ (**c**) t = 20 $\mathrm{\mu s}.$ (**d**) t = 30 $\mathrm{\mu s}$. (**e**) t = 50 $\mathrm{\mu s}.$

Taking the first scheme as an example, the forming process of a jet formed by three layers liner is shown in Fig. 3 below:

### Jet velocity analysis

**Figure 4:**
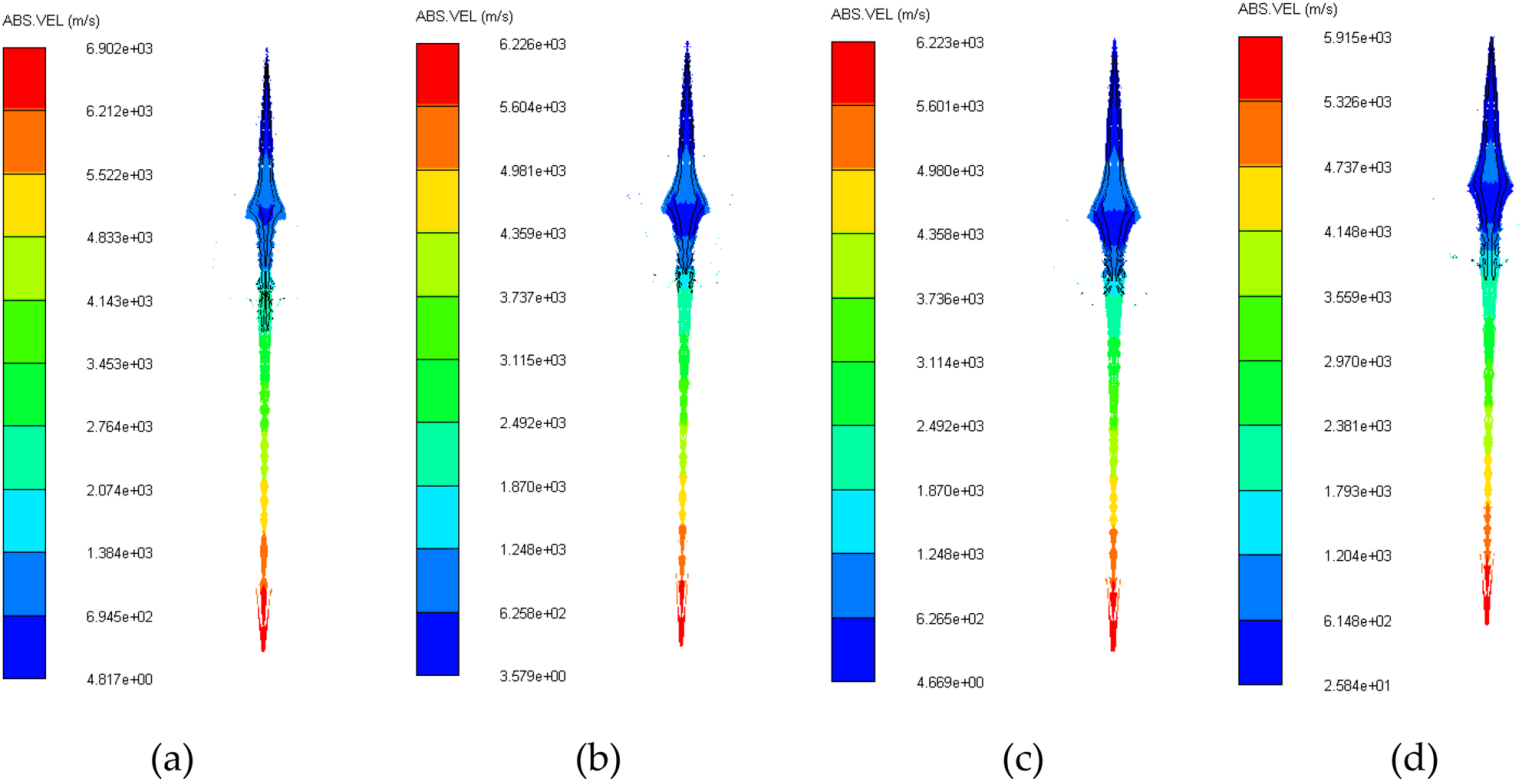
Jet velocity cloud diagram. (**a**) Scheme 1. (**b**) Scheme 2. (**c**) Scheme 3. (**d**) Scheme 4.

The comparative analysis of the jet velocity of each group of schemes can be analyzed by observing the velocity cloud map of the jet at the same time, and the overall velocity distribution of the jet can also be observed. The velocity contours of the four schemes in 50 μs jet forming are shown in Fig. 4^4,11–16^.

The jet head velocity of four different schemes in 50 μs jet forming is shown in Table 7 above. It can be seen from the above table that the jet head velocity of scheme 1 is the largest, and the jet head velocity of scheme 4 is the smallest.

**Table 7:** Velocity of jet head under different schemes (m/s).

Scheme	1	2	3	4
Head velocity	6902	6226	6211	5915

### Analysis of effective length of jet

After the explosive detonates and collapses the liner, a high-speed metal jet will be formed in the front of the jet, which is the main damage element of the penetration target, while the other part of the back will form a pestle, which is generally less than 1000 m/s, it has no great effect on penetration.

**Figure 5:**
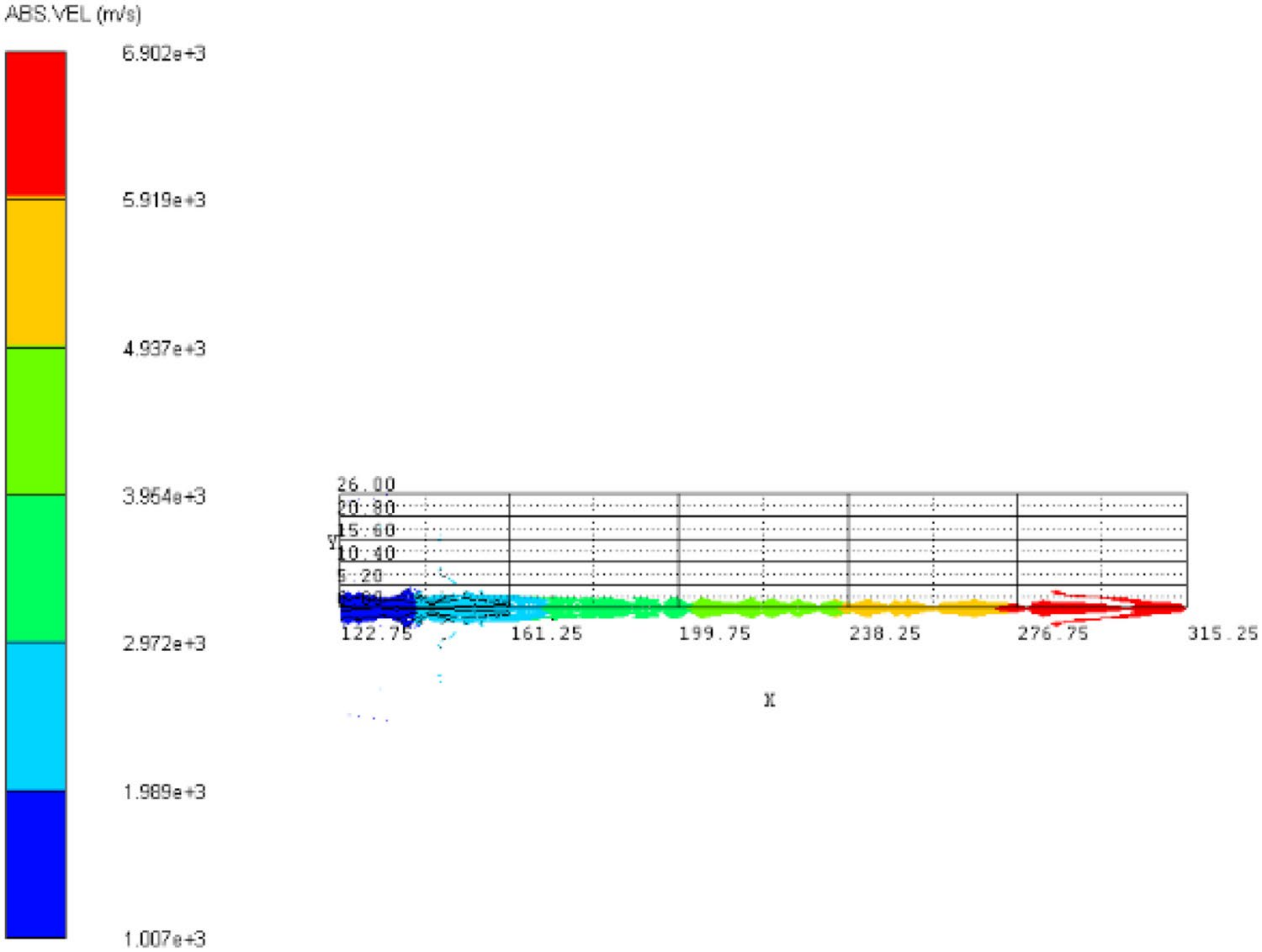
Jet effective length curve.

Taking scheme 1 as an example, the length of the part with the velocity above 1000 m/s is calculated to obtain the effective jet length, as shown in Fig. 5.

The Jet at the time of 50 μs was selected to calculate the effective length. The specific data of different schemes are shown in Table 8.

**Table 8:** Effective length of jet under different schemes (mm).

Scheme	1	2	3	4
Effective length	192.5	168.0	165.8	157.5

According to the analysis of effective jet lengths of different schemes in the table, scheme 1 has the longest effective jet length, while scheme 4 has the shortest effective jet length.

### Analysis of total jet energy

**Figure 6:**
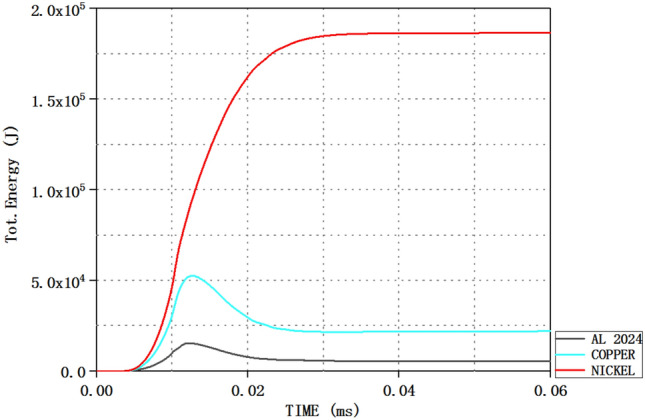
The energy change curve of different materials in the jet with time.

The larger the total energy of the jet is, the better the performance of the penetration target is. By selecting the scheme with the highest total energy, the optimal thickness ratio structure of the three-layer liner can be obtained by comparison. Therefore, the total energy is an important factor to measure the penetration performance of the jet. Taking scheme 1 as an example, AUTODYN software can be used to view the total energy of different materials in the three-layer liner. By calling the time-total energy diagram of different liner materials in AUTODYN software, the total energy value of different materials in the three-layer liner at the corresponding time can be obtained, as shown in Fig. 6.

Since the jet formed in this paper has three materials, Fig. 6 is the time-total energy curve of different liner materials. The total energy values of the three materials at different times can be obtained from the above figure. The total energy values corresponding to the three materials at 50 μs in Fig. 6 are added to obtain the total energy of the jet at this time. The total energy of the jet at 50 μs is shown in Table 9.

**Table 9:** Total jet energy under different schemes (kJ).

Scheme	1	2	3	4
Total energy	213.98	199.28	199.67	191.44

According to the total jet energy of different schemes in the table, the total jet energy of scheme 1 is the largest, while that of scheme 4 is the smallest.

### Analysis of total kinetic energy of jet

**Figure 7:**
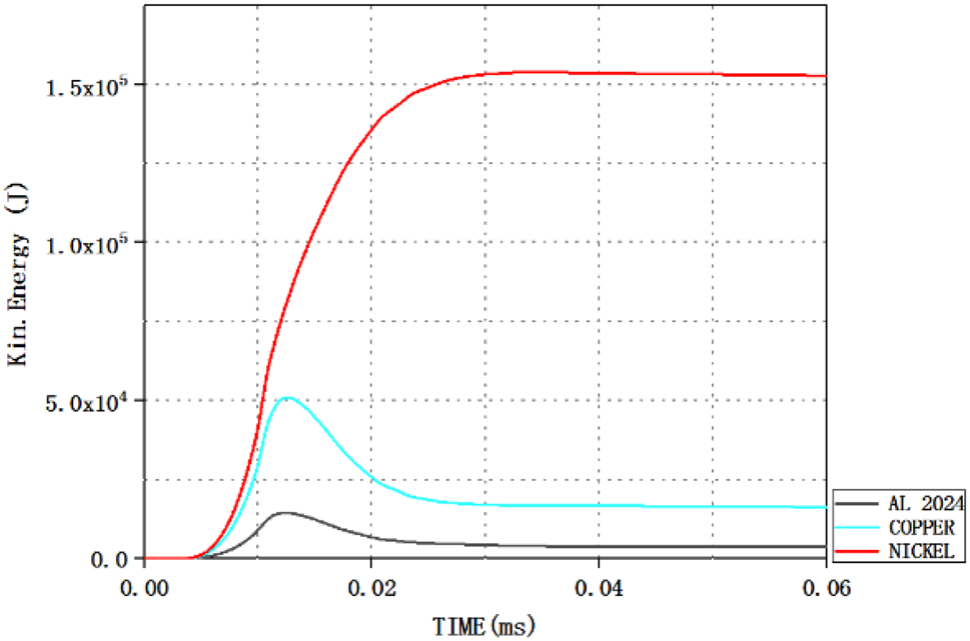
Kinetic energy curves of different materials in the jet flow with time.

The greater the total kinetic energy of the jet, the better the performance of the jet penetrating the target, so the total kinetic energy of the jet is also one of the important factors to measure the penetration performance of the jet. Taking scheme 1 as an example, AUTODYN software can be used to view the kinetic energy of different materials in the three-layer liner. By calling the time-kinetic energy diagram of different liner materials in AUTODYN software, the kinetic energy values of different materials in the three-layer liner at the corresponding time can be obtained, as shown in Fig. 7.

Since the jet formed in this paper has three materials, Fig. 7 is the time-kinetic energy curve of different liner materials. The kinetic energy values of the three materials at different times can be obtained from the above figure. The total kinetic energy of the jet at this time can be obtained by adding the kinetic energy values corresponding to the three materials at 50 μs in Fig. 7. The total kinetic energy of the jet at 50 μs is shown in Table 10.

**Table 10:** Total kinetic energy of jet under different schemes (KJ).

Scheme	1	2	3	4
Total kinetic energy	173.30	159.31	159.59	154.36

According to the total kinetic energy of Jet under different schemes in the table, the total kinetic energy of Jet in scheme 1 is the largest, while that in scheme 4 is the smallest.

### Effective jet mass analysis

The effective jet mass is an important index to measure the jet performance. By improving the effective jet quality, the utilization rate of the liner material can be improved. AUTODYN software can be used to view the mass of different materials in the three-layer liner, and call the relevant mass-time diagram. By calculating the total mass of the three materials at the time of 50 μs, and then calculating the total mass of the effective jet with a velocity of more than 1000 m / s at this time, the ratio of the effective jet mass to the total mass can be calculated. The effective jet mass of each group is shown in Table 11.

**Table 11:** Effective jet mass under different schemes (%).

Scheme	1	2	3	4
Effective jet mass	40.96	36.44	35.44	34.65

From the above table, it can be seen that when the outer and middle liner of the three-layer liner are fixed, the effective jet mass decreases with the increase of the material impact impedance of the inner liner; when the outer and inner liner of the three-layer liner are fixed, the effective jet mass decreases with the increase of the impact impedance of the middle liner material. When the middle and inner layer of the three-layer liner are fixed, the effective jet mass decreases with the increase of the impact impedance of the outer liner material. This shows that the inner liner is not a material with a larger impact impedance, and the jet performance is better. It is necessary to reasonably match the liner materials with different impact impedances to achieve the maximum material utilization and the best jet performance.

### comparative analysis

Material matching with different impact impedances has different influences on jet performance. For specific data, see Table 12 below.

**Table 12:** Jet performance parameters under different schemes (t = 50 μs).

Scheme	Head velocity (m/s)	Effective length (mm)	Total energy (KJ)	Total kinetic energy (KJ)	Effective jet mass (%)
1	6902	192.5	213.98	173.30	40.96
2	6226	168.0	199.28	159.31	36.44
3	6211	165.8	199.67	159.59	35.44
4	5915	157.5	191.44	154.36	34.65

From the analysis of the above simulation results, it can be seen that the jet head velocity, effective jet length, total jet energy, total jet kinetic energy and effective jet mass of scheme 1 are the largest.

It can be seen from the above table that when AL 2024 is the outer material, it is helpful to improve the head velocity, effective length and effective jet mass of the jet. When the impact impedance of the outer, middle and inner materials is increased respectively, the head velocity of the jet, the effective length of the jet and the effective jet mass are gradually reduced. When the outer and middle liner materials are unchanged and the impact impedance of the inner liner material is increased, the head velocity of the jet, the effective length of the jet and the effective jet mass are significantly reduced.

By observing the total energy and total kinetic energy of the four schemes, it can be seen that compared with scheme 1 and scheme 2, when the outer and middle liner materials are unchanged, the impact impedance of the inner liner material is increased, and the total energy and total kinetic energy of the jet are significantly reduced; compared with scheme 2 and scheme 3, when the outer and inner liner materials are unchanged, the impact impedance of the middle liner material is increased, and the total energy and total kinetic energy of the jet are only slightly increased. Compared with scheme 3 and scheme 4, when the middle and inner liner materials are unchanged, the impact impedance of the outer liner material is increased, and the total energy and total kinetic energy of the jet are slightly reduced. Compared with scheme 4, scheme 1, scheme 2 and scheme 3, as the outer liner material, AL 2024 is obviously better than copper; compared with scheme 2,3,4, nickel is superior to tantalum as the inner liner material in scheme 1.

In summary, the optimal material combination of the three-layer liner is scheme 1, which should be arranged in the order of AL 2024, copper and nickel from outside to inside.

## Effect of thickness ratio of three-layer liner on jet performance

Through the above research, it is proved that the jet performance is the best when the material of the three-layer liner is scheme 1. Therefore, on the basis of the material of the three-layer liner from the outside to the inside is AL 2024, copper and nickel, the optimal thickness ratio of the three-layer liner is studied, and a total of 10 groups of schemes are established. The total thickness of the three-layer liner is 3 mm, the model structure parameter data is unchanged, only the thickness ratio of the three-layer liner is changed, and the 10 groups of schemes are shown in Table 13.

**Table 13:** Thickness ratio scheme of three-layer liner.

Scheme	thickness ratio (Outer/middle/inner)	Scheme	thickness ratio (Outer/middle/inner)
1	1/1/4	6	2/2/2
2	1/2/3	7	2/3/1
3	1/3/2	8	3/1/2
4	1/4/1	9	3/2/1
5	2/1/3	10	4/1/1

**Figure 8:**
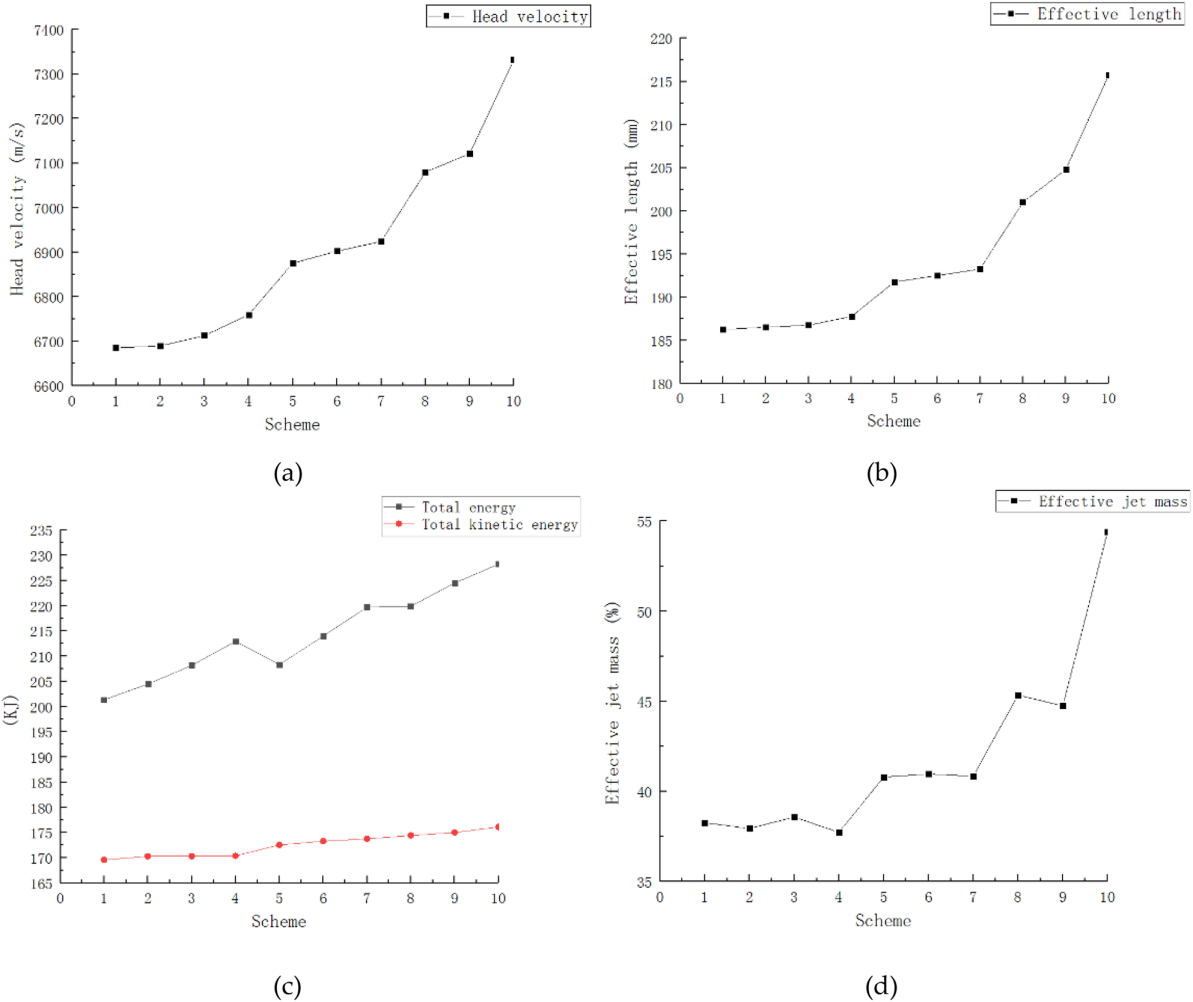
Jet performance parameters under different thickness ratios (t = 50 μs). (**a**) Jet head velocity under different schemes. (**b**) The effective length of jet under different schemes. (**c**) The total energy and total kinetic energy of the jet under different schemes. (**d**) Effective mass of jet under different schemes.

The influence of different thickness ratios in the three-layer liner on the jet performance is different. By setting 10 groups of different thickness ratios of the three-layer liner, the head velocity of the jet, the effective length of the jet, the total energy of the jet, the total kinetic energy of the jet and the effective jet mass are observed, and the optimal thickness ratio of the three-layer liner is obtained. The jet performance parameters generated by different thickness ratios of the three-layer liner are shown in Table 14 and Fig. 8.

**Table 14:** Jet performance parameters under different thickness ratios (t = 50 μs).

Scheme	Shape of jet	Head velocity (m/s)	Effective length (mm)	Total energy (KJ)	Total kinetic energy (KJ)	Effective jet mass (%)
1		6685	186.25	201.36	169.60	38.26
2		6689	186.50	204.47	170.29	37.93
3		6712	186.75	208.17	170.30	38.57
4		6759	187.75	212.96	170.38	37.72
5		6875	191.75	208.34	172.55	40.79
6		6902	192.50	213.98	173.30	40.96
7		6924	193.25	219.77	173.75	40.84
8		7080	201.00	219.88	174.42	45.34
9		7121	204.80	224.53	175.03	44.74
10		7332	215.75	228.27	176.12	54.40

It can be seen from Table 14 and Fig. 8a that as the thickness of the outer liner increases, the velocity of the jet head also increases. When the thickness ratio of the outer liner is the largest, the velocity of the jet head also reaches the maximum. The thickness ratio of the outer liner remains unchanged, and the thickness of the middle liner is gradually increased, and the head velocity of the jet only increases slightly. The thickness ratio of the outer liner remains unchanged, and the thickness of the inner liner is gradually increased, and the head velocity of the jet is gradually reduced. When the thickness of the middle or inner liners is kept constant, the velocity of the jet head will increase with the increase of the thickness of the outer liner. It shows that the key to affecting the velocity of the jet head is the thickness ratio of the outer liner.

It can be seen from Table 14 and Fig. 8b that the effective length of the jet increases with the increase of the thickness of the outer liner. When the thickness of the outer liner remains unchanged, the effective length of the jet remains basically unchanged. When the thickness of the outer liner remains unchanged, the effective length of the jet only increases slightly with the increase of the thickness of the middle liner. The thickness ratio of the outer liner remains unchanged, and the thickness of the inner liner is gradually increased, and the effective length of the jet is slightly reduced. Keeping the middle or inner liner unchanged respectively, increasing the thickness of the outer liner, the effective length of the jet will increase significantly.

It can be seen from Table 14 and Fig. 8c that when the thickness of the outer liner remains unchanged, the total energy and total kinetic energy of the jet only increase slightly with the increase of the thickness of the middle liner. When the thickness of the outer liner increases, the total energy and total kinetic energy of the jet keep an overall increasing trend. When the thickness of the outer liner accounts for the largest proportion, the total energy and total kinetic energy of the jet are the largest.

It can be seen from Table 14 and Fig. 8d that when the thickness of the middle liner remains unchanged, the thickness of the outer liner is gradually increased, and the effective mass of the jet is gradually increased. When the thickness of the inner liner remains unchanged, the thickness of the outer liner gradually increases, and the effective mass of the jet also gradually increases. When the thickness of the outer liner increases, the effective mass of the jet maintains an overall increasing trend, which indicates that the key to affecting the effective mass of the jet is the thickness of the outer liner.

In summary, the key to affecting the jet forming is the thickness ratio of the outer liner. Within a certain range, the larger the thickness ratio of the outer liner, the better the jet forming. Therefore, when the thickness ratio of the three-layer liner is 4/1/1 from the outside to the inside, the jet head velocity, the effective length of the jet, the total energy of the jet, the total kinetic energy of the jet and the effective jet mass are the largest, and the jet forming is the best.

## Study on jet penetration performance

For different types of liners, the optimum height is different. Under the same standoff, it is not enough to compare the penetration ability of different types of liners. In the simulation, several standoff are taken for penetration calculation of different types of liners, and the penetration performance of different types of liners on the target plate is obtained and compared.

### Numerical model establishment

In the analysis of the problem of shaped charge penetrating target, semi-infinite target is one of the commonly used target forms. In this paper, several standoff of the three-layer liner are used to calculate the penetration of the semi-infinite target plate. By studying the penetration depth and opening size of the jet to the semi-infinite target plate under different standoff, the performance parameters of the three-layer liner shaped charge penetrating the target plate are obtained. The specific parameters of the simulation are as follows: the main charge length of the warhead is 78 mm, the charge diameter is 60 mm, the cone angle of the liner is 60°, the shell thickness is 2 mm, the total thickness of the three-layer liner is 3 mm, and the material of the liner is AL 2024-copper-nickel from outside to inside. The length of the target plate model is 400 mm, the height is 50 mm, and the target plate material is steel 45. In order to effectively improve the utilization rate of the liner material and ensure that the inner metal is sufficient to form a penetrating jet, the thickness of the three-layer liner studied in this section is shown in Table 15 below. At the same time, the penetration ability of single-layer copper liner and double-layer AL 2024-copper liner with the same total thickness is compared and analyzed^17–22^.

**Table 15:** Thickness ratio scheme of liner.

Liner type	Scheme	Thickness ratio
Three-layer liner	a	1/1/4
	b	1/2/3
	c	2/1/3
Double-layer liner	d	0.5/2.5
	e	1/2
	f	1.5/1.5
Single-layer liner	g	1

**Figure 9:**
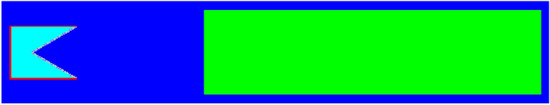
Finite difference model of jet penetrating target plate.

Taking scheme a as an example, the numerical calculation model of jet penetrating target formed by three-layer liner is shown in Fig. 9.

### Analysis of shaped charge penetrating target plate

**Figure 10:**
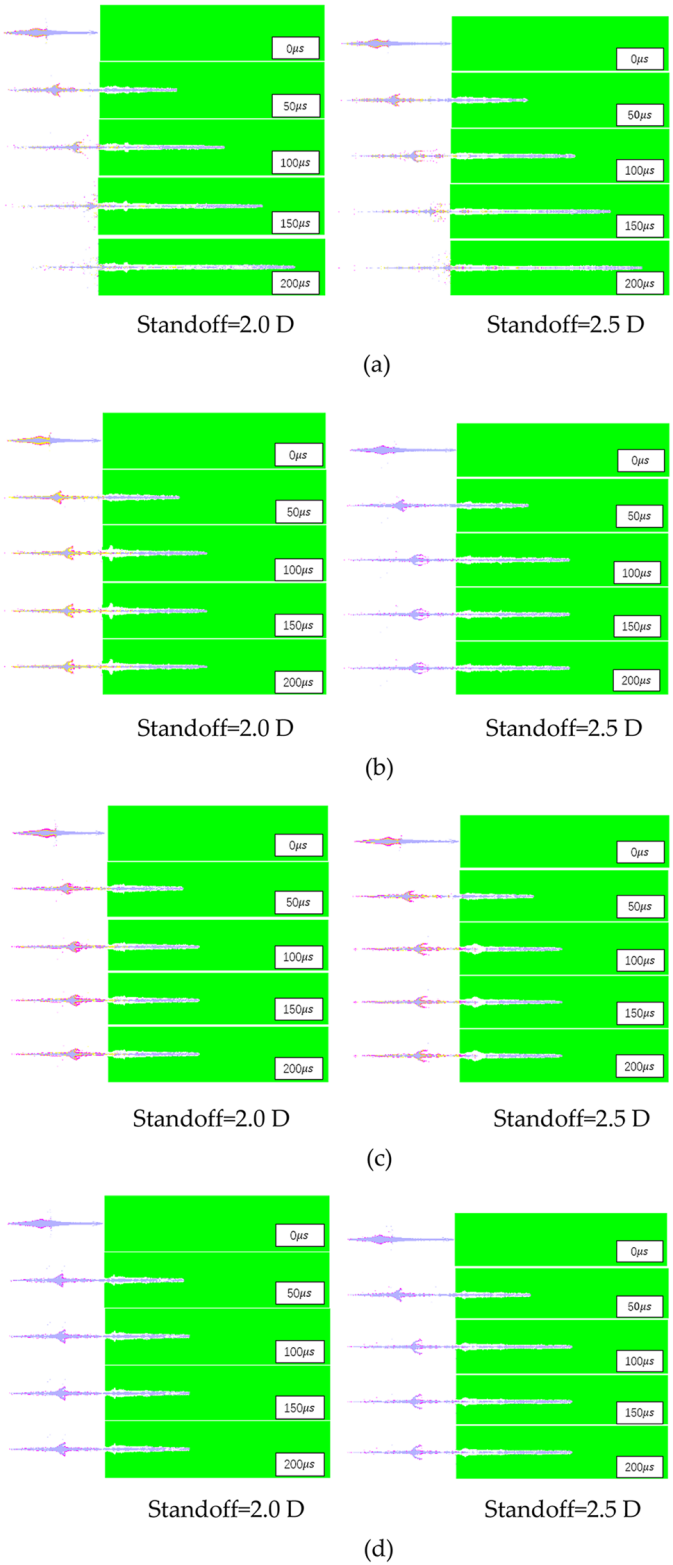

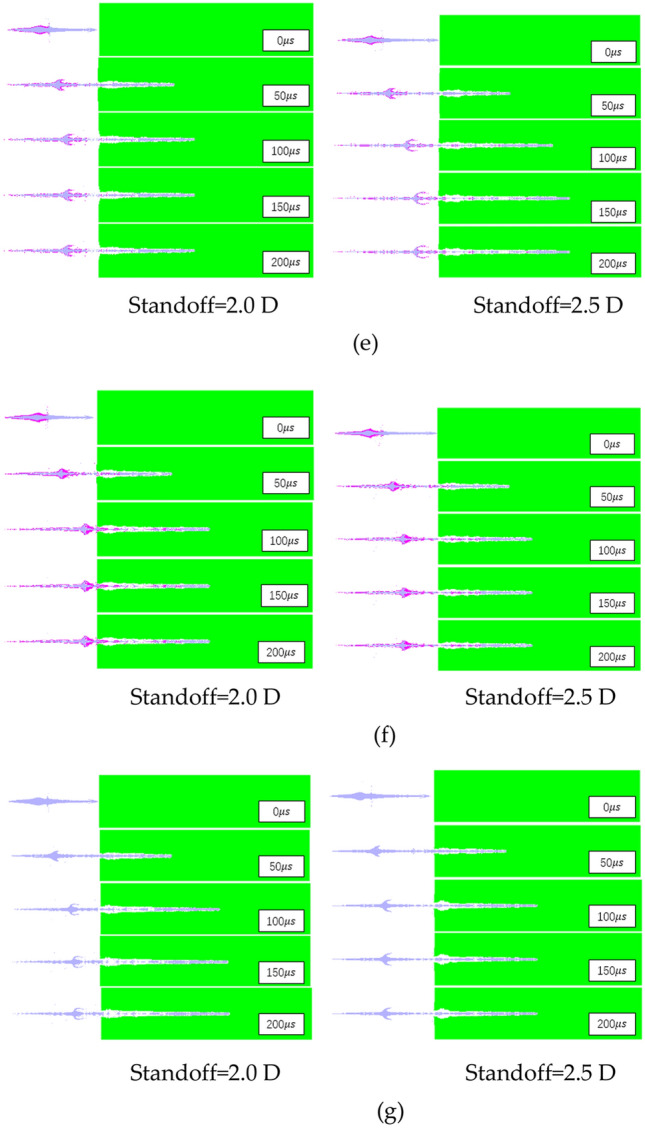
Penetration process of shaped charge.

**Figure 11:**
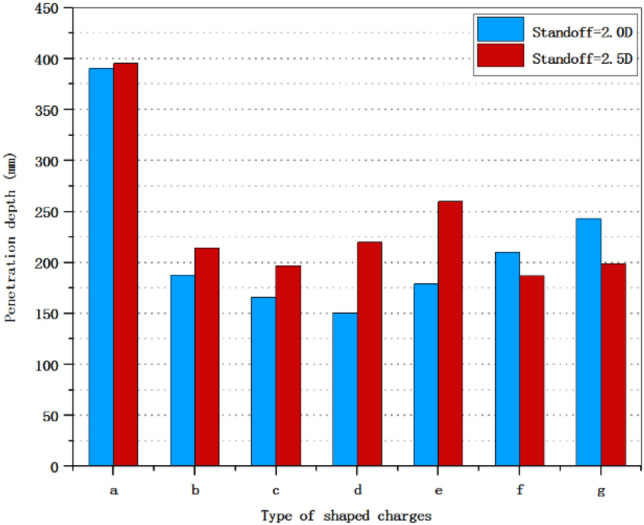
Penetration depths of shaped charge at 2.0 D and 2.5 D standoff distances.

**Figure 12:**
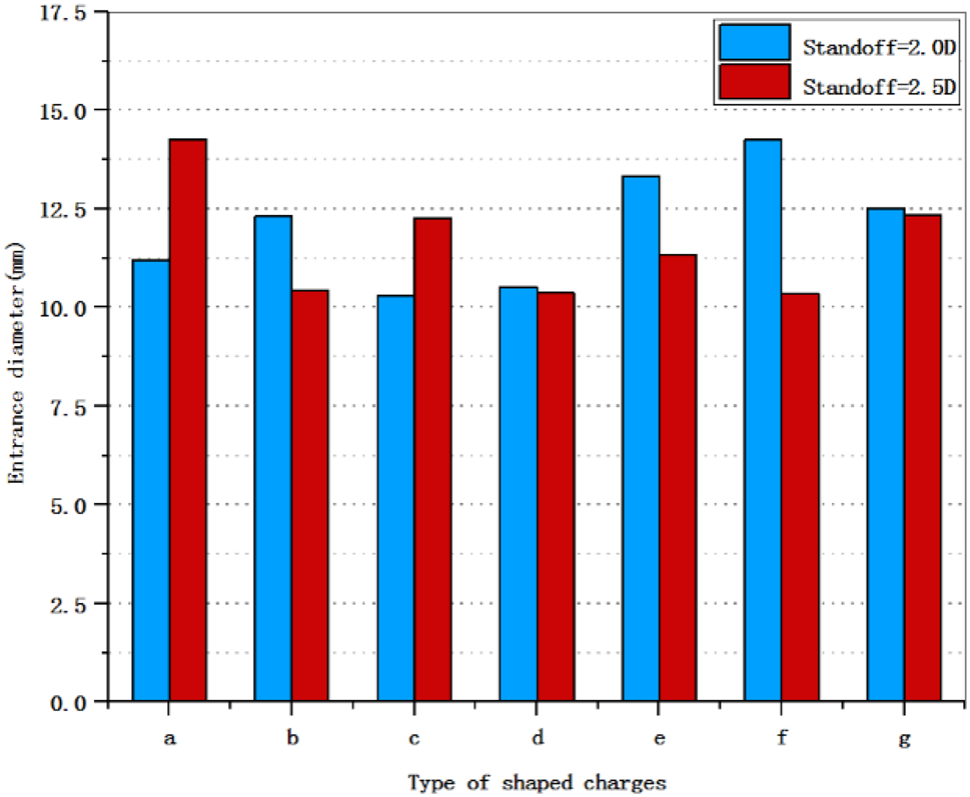
The entrance diameter of shaped charge at 2.0 D and 2.5 D standoff distances.

In order to explore the penetration ability of different types of liners to the target plate, the numerical calculation of jet penetration into the target plate is carried out. In order to avoid jet fracture, two smaller target distances of 2 D and 2.5 D are considered to explore its penetration ability. The penetration process of different types of liners is shown in Fig. 10 below:

The process of the three-layer liner penetrating the target plate is similar to that of the double-layer liner and the single-layer liner. It can be clearly seen that the opening stage, quasi-steady stage and termination stage of the jet. The penetration depth and hole size of shaped charge are shown in Figs. 11 and 12 below.

The parameters of the shaped charge penetrating the target plate at a distance of 2.0 D and 2.5 D are shown in Figs. 11 and 12. Figure 11 shows the penetration depth of all shaped charge in this paper. Obviously, the penetration depth of shaped charge with scheme a is much longer. The maximum penetration depth of scheme a is 395.5 mm; the maximum penetration depth of the double-layer liner is 259.7 mm when scheme e is adopted. The maximum penetration depth of the single-layer liner is 243.2 mm. Compared with the shaped charge with double-layer liner, the penetration depth of the shaped charge with scheme a is increased by 52.3%. Compared with the shaped charge with single-layer liner, the penetration depth is increased by 62.6%.

Figure 12 shows the entrance diameter of shaped charge in this paper. It can be seen from the figure that in the three-layer liner, the entrance diameter of the shaped charge with scheme a is the largest, which is 14.3 mm. The shaped charge with double-layer liner has the largest entrance diameter of 14.3 mm when scheme f is adopted, which is the same as the maximum entrance diameter of scheme a. Compared with the maximum entrance diameter of the shaped charge with a single-layer liner of 12.5 mm, the entrance diameter of the shaped charge with scheme a is increased by 14.4%.

## Conclusions

In this paper, a new three-layer liner structure is designed. By analyzing the matching of four kinds of liner materials with different impact impedance, the influence of the matching of the thickness ratio of the three-layer liner on the jet performance and the penetration of the semi-infinite target plate with steel 45, the following conclusions are drawn. This conclusion may only be applicable to the geometry of charge and liner studied in this paper, and may not be general, but it can provide some reference for the research of shaped charge warhead.

1. By comparing four kinds of liner materials with different impact impedance, the head velocity, effective length, total energy, total kinetic energy and effective jet mass of the jet formed when the three-layer liner material is AL 2024-copper-nickel from outside to inside are the best. Reasonable matching of liner materials with different impact impedance can achieve the maximum material utilization and the best jet performance.
2. In the analysis of the matching of the thickness ratio of the three-layer liner, the key to the jet forming is the thickness ratio of the outer liner. Within a certain range, the larger the thickness ratio of the outer liner, the better the jet forming.
3. The material of the three-layer liner is AL 2024-copper-nickel from the outside to the inside. When the thickness ratio of the liner is 4/1/1 from the outside to the inside, the jet head velocity, the effective length of the jet, the total energy of the jet, the total kinetic energy of the jet and the effective jet mass are the largest, and the jet forming is the best.
4. The maximum penetration depth of the shaped charge with a thickness ratio of 1/1/4 from the outside to the inside of the three-layer liner is 395.5 mm, which is 52.3% higher than that of the shaped charge with a double-layer liner. Compared with the shaped charge with single-layer liner, the penetration depth is increased by 62.6%.
5. When the thickness ratio of the three-layer liner is 1/1/4 from the outside to the inside, the maximum entrance diameter of the target plate is 14.3 mm, which is the same as that of the shaped charge with the double-layer liner. Compared with the shaped charge with single-layer liner, the entrance diameter is increased by 14.4%.

## Data Availability

The data used to support the findings of this study are included within the article.
